# Increased levels of circulating LPS during Tuberculosis prevails in patients with advanced pulmonary involvement

**DOI:** 10.1371/journal.pone.0257214

**Published:** 2021-09-10

**Authors:** Georgina Gallucci, Natalia Santucci, Ariana Díaz, Bettina Bongiovanni, Diego Bértola, Walter Gardeñez, Mauricio Rassetto, María Luisa Bay, Oscar Bottasso, Luciano D’Attilio

**Affiliations:** 1 Instituto de Inmunología Clínica y Experimental Rosario (IDICER), CONICET-UNR, Rosario, Santa Fe, Argentina; 2 Facultad de Ciencias Médicas, Universidad Nacional de Rosario, Rosario, Santa Fe, Argentina; 3 Servicio de Clínica Médica, Hospital Provincial del Centenario, Rosario, Santa Fe, Argentina; 4 Servicio de Neumología, Hospital Provincial del Centenario, Rosario, Santa Fe, Argentina; 5 Centro de investigación y biotecnología (CIBIO) Wiener Lab, Rosario, Argentina; Consejo Nacional de Investigaciones Cientificas y Tecnicas, ARGENTINA

## Abstract

Our earlier studies in tuberculosis (TB) patients indicate that in those where the process evolves to a larger pulmonary involvement, the immune endocrine response may promote an unfavorable environment. Chronic infectious diseases, and their persistent proinflammatory response, may affect mucosal barriers integrity favoring the translocation of gastrointestinal bacteria, leading to an increase of circulating lipopolysaccharides (LPS). Consequently, we quantified LPS levels in TB patients, with different degrees of pulmonary involvement, and controls (Co) and analyzed the possible relationship between LPS and inflammatory mediators i.e., C reactive protein (CRP), interleukin 6 (IL-6) and Interferon-gamma (IFN-γ), Erythrocyte Sedimentation Rate (ESR), steroid hormones (Cortisol and Dehydroepiandrosterone, DHEA), and inflammatory transcripts from peripheral blood mononuclear cells (IL-1β, IL-6, IFN-γ). LPS was assessed by the Limulus amoebocyte lysate assay and the ELISA technique was used to quantify hormones and cytokines in the plasma samples. Cytokine transcripts from PBMC were evaluated by qRT-PCR. Non-parametric tests were used. LPS levels were increased in TB patients, as did levels of CRP, IL-6, IFN-γ, cortisol and ESR. Severe patients had the highest amounts of circulating LPS; with moderate and severe cases showing much higher levels of CRP, ESR, IL-6, IFN-γ and cortisol/DHEA ratio, as an endocrine imbalance. Only in PBMC from severe cases was mRNA for IL-1β increased. Correlation analysis showed that levels of LPS from severe patients were positively associated with IL-6 and IFN-γ plasma concentrations and with IL-1β transcripts, while IL-6 had a positive correlation with the cortisol/DHEA ratio. The higher levels of circulating LPS during progressive TB may emerge as a contributing factor for the persistence of the greater immune endocrine imbalance distinctive of advanced disease, which might suggest a vicious cycle among LPS, inflammation and endocrine imbalance.

## Introduction

The bidirectional communication between the neuroendocrine and immune systems is a fact that medicine has recognized for a long time as revealed by a diverse series of studies both in humans and in experimental models. Many studies show that the products of the immune response modify the functioning of the neuroendocrine system, while a no lesser number of findings indicate, in turn, that hormones directly alter the activity of immune cells and therefore the course of diseases with inflammatory, autoimmune, or infectious background. This cross relationship between the immune and neuroendocrine systems is partly due to the stimulatory action of inflammatory cytokines on the hypothalamus-pituitary-adrenal (HPA) axis. Briefly, cytokines such as IL-6, IL-1β, and TNF-α stimulate the production of corticotrophin-releasing hormone in the hypothalamus with the subsequent release of adrenocorticotropin from the pituitary gland which in turn promotes the secretion of steroid hormones at the adrenal cortex [1, 2]. Regrettably, the implications of such tradeoffs in the medical field remains underappreciated. Our studies in the case of TB indicate that the immune-endocrine interrelationships are certainly relevant in pathophysiological terms [3, 4].

Tuberculosis, caused by *Mycobacterium tuberculosis*, essentially affects the lung, where it can produce from a few tissue lesions to a stage of intense inflammation with large parenchymal destruction. This variation in the degree of organic involvement seems to be related to the cellular immune response towards mycobacteria, which can mediate both protection and pathology. The essential elements participating in this response involve macrophages and T cells. The former phagocytose mycobacteria, promoting the production of different cytokines and antigen presentation to CD4^+^ T cells, which in turn synthesize Th1 type mediators, like IFN-γ and IL-2, capable of enhancing the bacteriostatic-bactericidal action of macrophages and promoting the proliferation of responding lymphocytes, respectively [5, 6]. While these responses are involved in the protective anti-mycobacterial immunity, in some instances Th1 immunity can also result in unbalanced pulmonary inflammation [7].

Within the context of the immune endocrine interactions, we showed that TB patients had reduced levels of dehydroepiandrosterone (DHEA), in presence of higher concentrations of proinflammatory mediators and cortisol, even further in patients with progressive disease along with a more unbalanced Cortisol/DHEA ratio [3, 4].

Studies from the immune and microbiological field suggest that exposure to stressful situations and the ensuing activation of the HPA axis, is likely to result in mucosal involvement with the consequent alteration of the epithelial barrier, and an increased possibility of bacterial translocation [8]. LPS plays an important role in the inflammatory response given its ability to trigger the phlogistic reaction by interacting with receptors present in a wide variety of cells, mainly the immune ones. In general, LPS activates the TLR4 mediated pathway leading to the activation of NF-κB followed by the secretion of pro-inflammatory cytokines such as TNF-α, IL-1β, and IL-6 [9, 10].

While studies in TB patients showed that they were more likely to present LPS in circulation [11, 12] the question remains as to whether the phenomenon bears some relationship with disease severity and the profile of immune-endocrine disturbances or the expression of genes encoding for inflammatory compounds. Our former studies indicate an increase of pro and anti-inflammatory cytokines according to disease progression, mirrored by an appreciable increase in inflammatory mediators in patients with mild disease, to higher levels of these cytokines among those with advanced disease [4]. As stated, differences according to disease severity also extend to the presence of adrenal steroids, wherein patients with advanced TB present a more unbalanced cortisol/DHEA ratio at the expense of increased and decreased cortisol and DHEA levels, respectively [13].

From the foregoing, newly diagnosed TB patients with different degrees of lung involvement were now assessed for the presence of LPS in circulation in addition to analyzing its relationship with mediators involved in the immune-endocrine response (proinflammatory cytokines and adrenal steroids), and inflammatory transcripts from PBMC.

## Materials and methods

### Study groups

A total of 38 TB patients was selected from newly diagnosed adult patients (18 to 65 years old). All patients had negative HIV serology, and the diagnosis of TB was based on clinical and radiological criteria along with the identification of acid-fast bacilli in sputum. The degree of involvement was classified, as in formerly studies [14], according to the extent and type of radiological lesion in: mild, patients with a single lung lobe involved without visible cavities; moderate, patients with unilateral involvement of 2 or more lobes and cavities, if present, not exceeding a total diameter of 4 cm; advanced or severe, bilateral disease with massive involvement and multiple cavities. Data were also collected concerning age and sex, as well as routine laboratory blood values, a history of alcoholism, or smoking-related chronic obstructive pulmonary disease. Exclusion criteria included, pregnancy, breastfeeding mothers, previous endocrine disorders, as well as treatment with corticosteroids, other hormones, immunosuppressive drugs, or immunomodulators. Controls (Co, n = 39) consisted of volunteers similar as to sex and age, and socioeconomic conditions with negative antecedents for contact with TB patients, immunological diseases, or endocrine disorders. Participants were enrolled once they had given their written informed consent to participate. All of them were previously informed about the study purposes, which were conducted according to the principles of the Declaration of Helsinki. The study was approved by the Ethics Review Committee from the Faculty of Medical Sciences, University of National Rosario (Ethics application number: 3157/2017).

### Sample blood collection and plasma isolation

Blood samples from TB patients were obtained immediately before the initiation of anti-tuberculosis treatment. Samples were collected at 8 a.m. to avoid differences due to circadian variations. An aliquot from blood samples was used to quantify the following clinical laboratory variables: complete blood count with platelet count, glycemia, hemoglobin A1C (HbA1C), urea, creatinine, uric acid, total proteins, albumin, aspartate aminotransferase (AST), Alanine Aminotransferase (ALT), alkaline phosphatase (ALP), serum cholinesterase, total cholesterol, HDL cholesterol, LDL cholesterol, triglycerides, and erythrocyte sedimentation rate (ESR). Plasma was obtained from fresh EDTA-treated blood. Samples were centrifuged at 2000 rpm for 30 min and plasma was collected and stored at -20°C.

### Blood sample PBMC isolation

After plasma isolation, PBMC was obtained from the buffy coat using Ficoll-Paque Plus gradient (density 1.077, Amersham Biosciences, NJ, USA) according to the manufacturer’s recommendations. The concentration and viability of PBMC were assessed using trypan blue staining in the Neubauer camera. Only samples with viability greater than 98% were used. Between 5 to 8 x 10^6^ cells/ml of TRIzol (Invitrogen, Carlsbad, USA) were stored at -80°C until mRNA extraction.

### Assessment of cytokines, hormones, and CRP in plasma

The levels of IFN-γ (BD Pharmingen, detection limit-DL: 4.7 pg/ml), IL-6 (BD Pharmingen, detection limit DL: 3.9 pg/ml) and hormones like cortisol (DRG Diagnostics, DL: 2.5 ng/ml) and DHEA (DRG Diagnostics, DL: 0.129 ng/ml) were assayed by commercial enzyme-linked immunosorbent assays according to the manufacturer’s recommendations.

C reactive protein (CRP) levels were measured by using a high sensitivity automatic analyzer by turbidimetric test (Wiener Lab, Rosario, Argentina, DL: 2.5 mg/l).

### Assessment of plasma LPS

Plasma LPS levels were evaluated using the Hycult chromogenic endpoint LPS detection assay (Limulus amoebocyte lysate assay, Hycult Biotech, Uden, The Netherlands) according to the manufacturer’s instructions. The kit has a minimum detection limit of 0.04 EU/ml.

### RNA isolation, cDNA synthesis and qPCR

Total RNA was isolated from PBMC, obtained as described in section 2.3, using TRI reagent (Genbiotech) according to the manufacturer’s instructions. cDNA was synthesized from 2 μg of total RNA by extension of oligo-dT primers with M-MuLV reverse transcriptase (Thermo Fisher). qPCR was performed with the StepOnePlus (96 well) Real-Time PCR Systems (Applied Biosystems) using 3 μl of cDNA dilution, 0,4 μM of each primer and 3 μl of 5x HOT FIREPol® EvaGreen qPCR Mix Plus (ROX) (Solis BioDyne), the final volume of 15 μl. Thermal cycling conditions were as follows: 10 min at 95°C followed by 40 PCR cycles of denaturing at 95°C for 7s, 25s for annealing at 60°C and 30s for elongation at 72°C. Fluorescence readings were performed during 10s at 80°C before each elongation step. To normalize the expression of every gene, the transcript of peptidylprolyl isomerase A (PPIA) was used as an endogenous control on each mononuclear cell sample [15]. Serially diluted cDNA samples were used as relative external standards curve in each run. This allows performing “The Relative Standard Curve Method” for the relative quantification of gene expression, as performed formerly [16]. Similarity and homogeneity of PCR products from samples were confirmed by automated melting curve analysis (StepOne Software, Applied Biosystems). The primers used are described in Table 1. Data were expressed as an arbitrary unit (AU): fold change of the relative expression levels of the gene of interest normalized by the relative expression levels of reference gen PPIA.

**Table 1:** List of primer sequences used for qPCR analysis in this study.

Gene	Primer sequence	Product size (bp)
**PPIA** Gene ID:5478	**F:** 5’-GCATACGGGTCCTGGCATCTTG-3’	101
	**R:** 5’-TGCCATCCAACCACTCAGTCTTG-3’	
**IFN-γ** Gene ID: 3458	**F:** 5’-AACGAGATGACTTCGAAAAGCTG-3’	158
	**R:** 5’-TCTTCGACCTCGAAACAGCA-3’	
**IL-6** Gene ID: 3569	**F:** 5’-ACTGGTCTTTTGGAGTTTGAGGT-3’	191
	**R:** 5’-GTTGGGTCAGGGGTGGTTATTG -3’	
**IL-1β** Gene ID: 3553	**F:** 5’-TCTGTACCTGTCCTGCGTGTTG -3’	157
	**R:** 5’-GGGGAACTGGGCAGACTCAA -3’	

**F:** Forward primer. **R:** reverse primer. **PPIA: **peptidylprolyl isomerase A; **IFN-γ:** interferon-gamma; **IL-6:** interleukin-6; **IL-1β:** interleukin-1beta

### Statistical analysis

Comparisons among groups were performed by non-parametric methods, like the Kruskal Wallis analysis followed by a post-hoc test when applicable, for the multiple comparison approach. Correlations between cytokine, CRP, and LPS levels were analyzed by Spearman’s rank test. Categorical variables were compared by the chi-square test or Fisher´s exact test. Data were regarded as statistically significant whenever p<0.05.

## Results

### Characteristics of study participants

The main features of the sampled individuals are shown in Table 1. No major differences were seen regarding age, sex distribution and alcohol consumption. However, a significantly decreased body mass index was found in patients with TB compared to Co (p<0.0001). The same was true when comparing according to disease severity (Table 2). The number of smokers was more prevalent in TB patients (p<0.01).

**Table 2:** Characteristics of control subjects and TB patients.

	Co (n = 39)	TB (n = 38)	Mild (n = 6)	Moderate (n = 19)	Severe (n = 13)
**Age (years)**	32 (24–49)	34 (24–49)	38 (26–59)	32 (22–58)	34 (24–44)
**BMI (kg/m** ^**2**^ **)**	26.4 (24.5–29.4)	20.0 (18.6–21.3)*	19.7 (19.0–22.6)*	20.8 (19.4–24.4)*	19.5 (18.3–20.0)*
**SEX (F/M)**	12/27	12/26	3/3	5/12	4/11
**BCG (%)**	100	89.2	83.3	87.5	93.3
**Current smoker (Yes/No)**	7/29	19/19^#^	2/4	8/11	9/4^#^
**Alcohol consumption (Yes/no)**	7/29	12/26	3/3	4/15	5/8

Quantitative values are presented as median (interquartile range). Quantitative comparisons were performed by non-parametric methods, like the Kruskal Wallis analysis followed by a post-hoc test when applicable, for the multiple comparison approach.

* Represents statistically significant differences from Co (p<0.0001).

# Different from Co (Fisher’s exact test) p<0.01.

ns: not significant

When analyzing the results of the laboratory biochemistry, TB patients had significantly augmented neutrophils, and platelet counts along with decreased hemoglobin and lymphocyte values (Table 3), particularly the ones with moderate and severe disease. Only, in the group of severe patients, a lower number of erythrocytes was found when compared to the control group.

**Table 3:** Differential blood count.

Parameters [Reference value]	Co (n = 39)	TB (n = 38)	Mild (n = 6)	Moderate (n = 19)	Severe (n = 13)
**WBC (10**^**3**^**/μl)** [4–9]	6.1 (5.3–7.2)	8.7 (7.5–11.1)*	8.7 (6.2–13.7)	8.5 (7.5–9.5)*	11.0 (7.8–12.0)*
**RBC (M/μl)** [3.7–5.5]	4.7 (4.5–5.1)	4.6 (4.1–5.1)	4.3 (4.0–5.0)	4.9 (4.3–5.2)	4.3 (4.0–4.7)*
**HGB (g/dL)** [11–16]	14.2 (13.3–14.8)	12.4 (11.1–13.5)*	13.0 (11.5–13.3)	12.9 (11.4–14.1)*	12.0 (10.4–12.5)*
**Neutrophil (10**^**3**^**/μl)** [2–6]	3.2 (2.7–4.0)	5.8 (4.4–7.8)*	5.1 (3.9–9.8)*	5.4 (4.1–6.2)*	7.6 (6.1–8.6)*
**Lymphocyte (10**^**3**^**/μl)** [1–3]	2.0 (1.7–2.5)	1.6 (1.2–2.2)*	1.7 (1.2–2.5)	1.6 (1.3–2.2)*	1.4 (0.9–1.6)*
**Monocyte (10**^**3**^**/μl)** [0–1]	0.5 (0.4–0.6)	0.8 (0.6–1.0)*	0.8 (0.5–1.7)	0.7 (0.5–0.9)*	1.0 (0.6–1.4)*
**Eosinophil (10**^**3**^**/μl)** [0–0.4]	0.13 (0.09–0.19)	0.16 (0.10–0.21)	0.15 (0.12–0.18)	0.15 (0.10–0.20)	0.16 (0.05–0.23)
**Basophil (10**^**3**^**/μl)** [0–0.09]	0.03 (0.02–0.05)	0.03 (0.02–0.05)	0.05 (0.02–0.06)	0.05 (0.03–0.05)	0.02 (0.01–0.04)
**Platelets (10**^**3**^**/μl)** [150–400]	229 (198–278)	431 (317–512)*	415 (253–641)*	447 (334–498)*	370 (307–537)*

Data are expressed as median (Interquartile range).

Quantitative comparisons were performed by non-parametric methods, like the Kruskal Wallis analysis followed by a post-hoc test when applicable, for the multiple comparison approach.

* Represents statistically significant differences from Co (p<0.05).

Co: controls; TB: pulmonary tuberculosis; WBC: White blood cells. RBC: Red blood cells; HGB: Hemoglobin

Compared to Co, TB patients revealed no significant differences in relation to the serum levels of glucose, uric acid, aspartate aminotransferase, alanine aminotransferase, and triglycerides although they exhibited increased amounts of HBA1c (Table 4). TB patients preferably the moderate ones had decreased urea, albumin, and HDL cholesterol concentration (Table 4). Also, a decline in albumin and serum cholinesterase was found in mild TB patients. Patients with advanced disease showed lower levels of creatinine, albumin, serum cholinesterase, total cholesterol, HDL and LDL cholesterol, and increased levels of alkaline phosphatase with respect to Co (Table 4).

**Table 4:** Blood biochemical parameters.

	Co (n = 39)	TB (n = 38)	Mild (n = 6)	Moderate (n = 19)	Severe (n = 13)
**Glycemia (mg/dl)** [70–100]	89 (82–93)	86 (82–95)	88 (80–97)	86 (83–93)	87 (81–96)
**Hemoglobin A1c (%)** [4.8–5.9]	5.2 (4.7–5.6)	5.5 (5.1–5.8)*	4.9 (4.6–5.7)	5.7 (5.4–5.8)*	5.6 (5.1–6.2)
**Urea (mg/dl)** [10–50]	30 (26–36)	22 (19–30)*	24 (14–34)	21 (20–28)*	23 (18–32)
**Creatinine (mg/dl)** [0.5–0.9]	0.8 (0.7–0.9)	0.7 (0.6–0.8)*	0.6 (0.6–0.9)	0.7 (0.6–0.9)	0.7 (0.6–0.9)*
**Uric Acid (mg/dl)** [2.4–5.7]	4.8 (3.9–5.6)	5.0 (3.8–8.3)	7.7 (4.0–9.2)	6.3 (3.8–9.3)	4.5 (3.6–4.9)
**Total Protein (g/dL)** [6.6–8.7]	7.4 (7.1–7.6)	7.9 (7.4–8.3)*	7.8 (7.4–8.1)	8.0 (7.7–8.3)*	7.8 (6.9–8.4)
**Albumin (g/dl)** [3.4–4.8]	4.5 (4.3–4.7)	3.9 (3.5–4.2)*	3.7 (3.6–4.0)*	4.0 (3.8–4.3)*	3.3 (3.1–3.9)*
**AST (UI/I)** [10–38]	19 (16–22)	16 (13–24)	17 (13–33)	15 (13–22)	18 (15–26)
**ALT (UI/I)** [10–41]	18 (12–23)	18 (12–24)	13 (4–15)	19 (12–26)	23 (14–26)
**ALP (U/I)** [35–104]	67 (53–76)	92 (83–109)*	87 (77–103)	85 (80–99)*	109 (93–198)*
**Serum cholinesterase (UI/I)** [6400–15500]	9157 (7911–10393)	6130 (5361–7202)*	5748 (5647–6663)*	6669 (5921–7452)*	4698 (3379–6568)*
**Total Cholesterol (mg/dl)** [50–200]	179 (152–193)	144 (121–177)*	181 (107–245)	145 (133–180)	124 (112–151)*
**HDL Cholesterol (mg/dl)** [40–100]	51 (45–61)	36 (25–44)*	43 (32–58)	35 (31–44)*	26 (18–39)*
**LDL Cholesterol (mg/dl)** [4–100]	107 (91–123)	88 (72–108)*	110 (66–142)	83 (72–108)	85 (73–98)*
**Triglycerides (mg/dl)** [50–150]	91 (61–99)	79 (69–102)	80 (70–124)	86 (69–97)	72 (66–103)

Data are expressed as median (Interquartile range). Quantitative comparisons were performed by non-parametric methods, like the Kruskal Wallis analysis followed by a posthoc test when applicable, for the multiple comparison approach.

* Represents statistically significant differences from Co (p<0.05). Co: controls; TB: pulmonary tuberculosis; AST: Aspartate Aminotransferase, ALT: Alanine Aminotransferase, ALP: alkaline phosphatase, HDL: high-density lipoproteins and LDL: Low-density lipoprotein.

**Fig 1:**
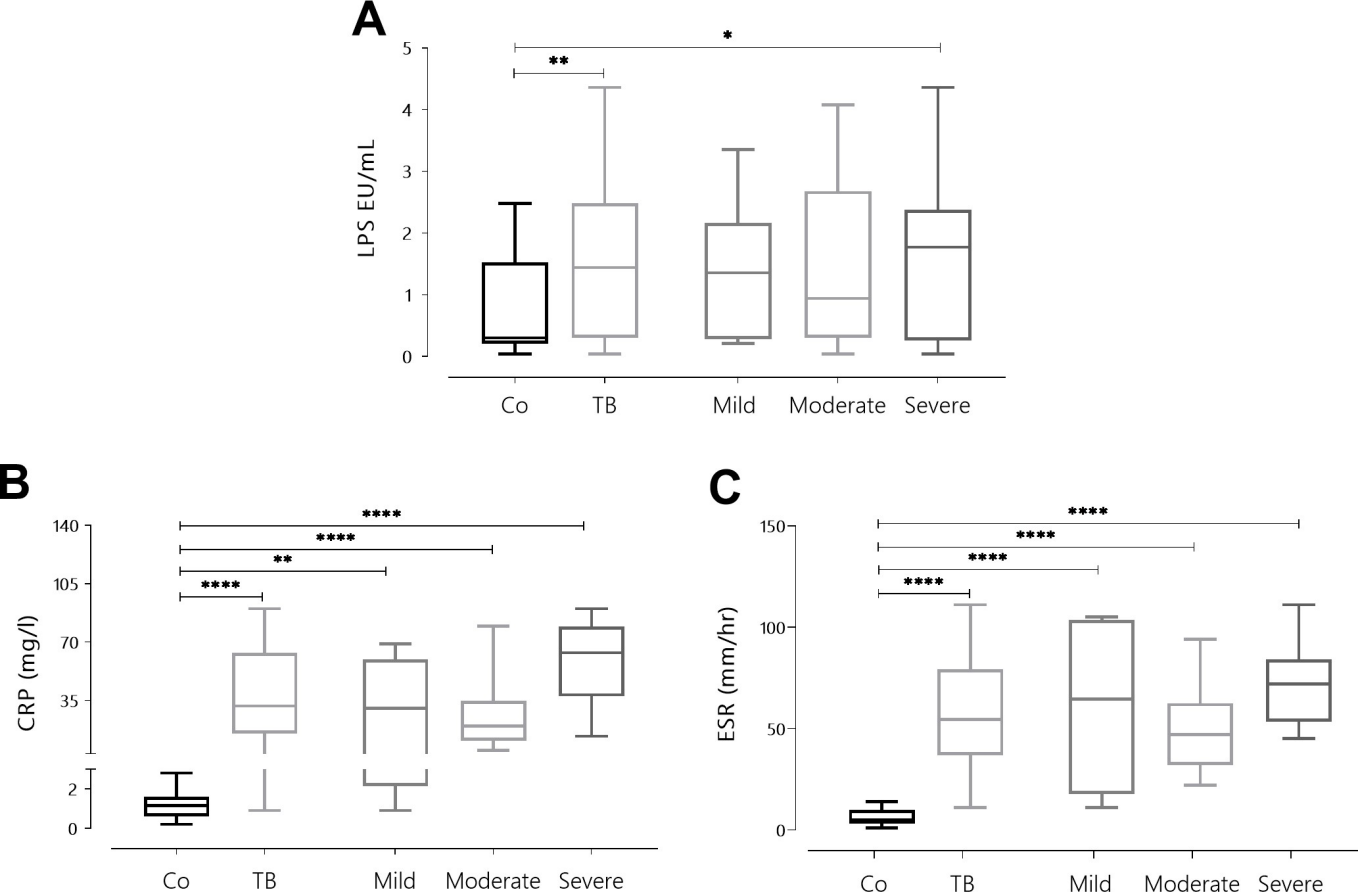
Plasma levels of Lipopolysaccharides (LPS) (A), C-reactive protein (CRP) (B) along with erythrocyte sedimentation rate (ESR) (C) in Co and TB patients. Box plots show median values, 25–75 percentiles from data in each group with maximum and minimum values. Statistically significant at *p <0.05, ** p <0.01, ***p <0.001, ****p <0.0001.

Comparison of LPS plasma levels, revealed that TB patients had higher amounts than Co (p<0.05), particularly in cases with severe disease (Fig 1A, p<0.01). It was clear that TB patients also showed elevated levels of CRP (Fig 1B, p<0.0001) and ESR (Fig 1C, p<0.0001), if compared to Co. Further comparisons indicated that the higher the amount of lung involvement the greater the PCR (p<0.005), and ESR values (p<0.0001).

In the same direction, the whole group of TB patients displayed augmented concentrations of IL-6 and IFN-γ (p<0.0001 and p = 0.0003, vs Co), as did moderate and severe cases (p<0.003 in both cases, Table 5). As depicted in the same Table, TB patients presented modestly increased levels of cortisol (p = 0.03) particularly the moderate ones (p = 0.01), whereas DHEA levels appeared quite lowered (p = 0.008, overall TB group) at the expense of severe TB cases (p = 0.0001 vs. Co). Such alterations resulted in an increased Cortisol/DHEA ratio in TB patients, statistically significant when compared with the one seen in Co (Mild, p = 0.006, Moderate, p = 0.0001, Severe, p = 0.004). When analyzing the gene expression for proinflammatory cytokines, severe patients had increased IL-1β mRNA levels (Table 5) with no between-group differences as to IL-6 and IFN-γ transcripts.

**Table 5:** Proinflammatory cytokines, adrenal hormones plasma levels and Cortisol/DHEA ratio together with mRNA levels for proinflammatory cytokines of PBMC from controls (Co), and patients with pulmonary tuberculosis (TB) with different degree of pulmonary involvement (mild, moderate and severe).

	Co	TB	Mild	Moderate	Severe
IL-6 [pg/ml]^§^	9.0 (6.4–10.6)	31.4 (20.9–42.4)*	18.2 (6.2–40.6)	25.7 (22.6–37.4)*	37.4 (21.7–63.5)*
IFN-γ [pg/ml]^§^	7.1 (5.1–14.0)	14.6 (9.0–23.8)*	11.9 (5.5–15.7)	13.7 (8.8–23.5)*	22.8 (14–34.6)*
IL-6 mRNA AU	0.28 (0.18–0.52)	0.28 (0.15–0.46)	0.18 (0.12–0.96)	0.30 (0.14–0.43)	0.29 (0.17–0.50)
IFN-γ mRNA AU	2.5 (1.1–3.2)	2.03 (1.20–3.27)	1.81 (1.35–2.24)	2.16 (1.55–3.56)	1.76 (0.65–3.82)
IL-1β mRNA AU	0.04 (0.01–0.11)	0.07 (0.02–0.36)	0.02 (0.01–0.03)	0.07 (0.02–0.24)	0.23 (0.04–0.58)*
Cortisol [ng/ml]**	195 (108–251)	213 (187–273)*	193 (174–218)	238 (208–304)**	189 (183–231)
DHEA [ng/ml]***	6.3 (3.8–8.4)	3.3 (2.4–6.2)*	4.1 (2.6–6.7)	4.6 (2.7–6.5)	2.4 (1.4–4.3)*
Cortisol/ DHEA^**#**^	26 (19–40)	55 (39–80)*	58 (40–81)	52 (36–77)*	66 (45–148)*

IL-6: interleukin-6; IFN-γ: interferon-gamma; IL-1β: interleukin-1beta; DHEA: Dehydroepiandrosterone; AU: Arbitrary Unit (fold change of the relative expression levels of the gene of interest normalized by the relative expression levels of reference gen PPIA).

Quantitative comparisons were performed by non-parametric methods, like the Kruskal Wallis analysis followed by a posthoc test when applicable, for the multiple comparison approach.

^§^Controls significantly lower than the whole group of TB patients (IL-6: p<0.0001, and IFN-γ: p = 0.0003), as well as moderate and severe cases (p<0.003, for in both cytokines).

*Significantly different from Co (p<0.0001).

**Controls significantly lower than the whole group of TB patients (p = 0.03) and moderate cases (p = 0.01).

***Controls significantly higher than the whole group of TB patients (p = 0.008) and severe cases (p = 0.0001).

^#^ Controls significantly lower than the whole group of TB patients (p<0.0001), mild (p = 0.006), moderate (p = 0.0001), and severe cases (p = 0.004).

Pairwise correlation analysis among TB patients revealed a positive or negative association between LPS with IL-6 and IFN-γ, or between LPS and DHEA, respectively (Table 6). In the case of Co, LPS was positively correlated with the Cortisol/DHEA ratio (r = 0.44, p<0.01). Among severe cases, LPS concentrations were positively associated with IL-6 and IFN-γ plasma levels as well as with PBMCs IL-1β transcripts.

**Table 6:** Correlation analysis of hormone, cytokine and LPS plasma levels in the overall group of TB patients and those with severe disease.

Pairwise correlations	TB overall group (n = 38)		Severe TB cases (n = 13)	
	r coefficient	p-value	r coefficient	p-value
LPS vs. IL-6		ns	0.59	0.03
LPS vs. IFN-γ		ns	0.80	0.01
LPS vs. IL-1β transcripts		ns	0.65	0.03
LPS vs. DHEA	-0.41	0.02		ns
CRP vs. Cortisol/DHEA	0.37	0.03		ns
IL-6 vs. Cortisol/DHEA		ns	0.64	0.03

ns: not significant

LPS: Lipopolysaccharides; IL-6: interleukin-6; IFN-γ: interferon-gamma; IL-1β: interleukin-1beta; CRP: C-reactive protein.

## Discussion

LPS is a constituent from the membrane of Gram-negative bacteria with potent immune-activating effects via the recognition by TLR-4 on immune cells leading to the production of proinflammatory mediators including CRP, and proinflammatory cytokines [17]. Diseases of chronic nature are accompanied by a persistent state of low-grade inflammation. This may help to explain the hematological and clinical biochemistry findings in TB patients, along with their increased amounts of HBA1c likely compatible with some degree of insulin resistance known to occur in TB [18]. Added to it, chronic inflammation is known to result in some alteration of the mucosal barriers and commensal bacteria that line the gastrointestinal tract favoring the translocation of bacteria to circulation and the ensuing presence of circulating LPS in modest concentrations (1–100 pg/ml). As such, LPS can be found in circulation in situations affecting the integrity of the intestinal mucosa, and hence promoting the translocation of microorganisms [17, 19].

In the present work we provide evidence demonstrating that plasma LPS levels were increased in TB patients, as did levels of inflammatory mediators (CRP, IL-6, IFN-γ) and the ESR. Further analysis according to TB severity, revealed that severe patients had the highest amounts of circulating LPS; with moderate and severe cases showing much higher levels of CRP, ESR, IL-6, and IFN-γ. Besides the involvement in the antimycobacterial response, these compounds are also related to the extent of pulmonary disease [20, 21], for which their increased presence during advanced disease may be more compatible with an inflammatory role, as is the case of increased IL-1β transcripts. Within the context, that the degree of microbial translocation measured by the assessment of systemic levels of LPS is related to the extent of intestinal permeability [22, 23], our study suggests an alteration at the mucosal level. Individuals with tobacco exposure are more likely to present modestly increased levels of LPS in circulation [24–26]. In the present series, the whole group of TB patients and particularly the severe cases had a higher frequency of the smoking habit. The latter is not only associated with an impaired intestinal barrier from the small bowel [27, 28] but also with cavitary lung lesions and decreased IFN-γ response [29, 30].

The increased levels of LPS may add another element to the array of factors accounting for the accompanying inflammation seen in TB patients, especially those with progressive disease. This also extends to several neuro-immune endocrine alterations we have seen in them [13, 14, 31], like modest increases of cortisol and quite reduced amounts of DHEA, resulting in an increased Cortisol/DHEA ratio. In addition to the fact that the moderate group of patients had a greater number of cases, their higher cortisol levels may be indicating that the relative deficiency of the HPA axis is less manifest in them. Unlike severe patients in whom cortisol levels remain within the normal range in presence of increased circulating amounts of proinflammatory compounds. Despite this, they present the highest cortisol/DHEA ratio. Beyond this assumption, the unbalanced cortisol/DHEA relation was shown to be related to an inhibition of *in vitro* cell-mediated immune responses, disease severity and inflammatory status [32]. Extending these findings, the present series of advanced patients had the highest cortisol/DHEA positively associated with IL-6 levels, as well. Moreover, the negative relationship between LPS and DHEA also provides another piece of evidence linking inflammation with decreased presence of this androgen.

There is evidence that in situations of prolonged inflammation, the increased and persistent production of IL-6 is likely to result in gut damage and the ensuing microbial translocation [33]. In the same sense IFN-γ and TGF-β may also alter mucosal permeability [34], as it may exert stressful situations [35] implying that these compounds may be also accounting for mucosal alterations. Being this the case, a sort of kind of vicious circle may ensue; that is the passage of LPS into the circulation which in turn increases the production of inflammatory mediators, further stimulating cortisol production and the ensuing mucosal alteration favoring the perpetuation of tissue damage. LPS is well-known for its stimulating effects on proinflammatory cytokine production [36, 37]; whereas CRP production is known to be increased upon challenge with Gram-negative bacteria [38, 39] or purified LPS [40]. Supporting this view our studies revealed a positive association between levels of LPS and IL-6 and IFN-γ, together with the IL-1β expression in PBMC.

As seen in other diseases of chronic nature, cirrhosis [41], diabetes [42], cardiovascular disorders [43], chronic infection and aging [44], two former studies also reported increased LPS levels in serum from patients with pulmonary or extrapulmonary TB [11]. By categorizing patients according to disease severity, we now extend this finding in the sense that such abnormality is more likely to prevail in advanced disease in presence of a more unbalanced immune-endocrine relationship.

## Conclusions

This study reveals that TB patients, preferably those with advanced disease, are more likely to present increased circulating amounts of LPS in coexistence with higher levels of proinflammatory mediators and cortisol/DHEA ratio, together with an increased expression of IL-1β in PBMC, consistent with a sort of kind of perpetuating process. Raised systemic levels of LPS may emerge as a contributing factor for the persistent immune-endocrine imbalance and chronic inflammation which underlies the pathogenesis of progressive TB.

## Supporting information

S1 File Database. (XLSX)
